# Spatiotemporal gait characteristics in patients with COPD during the Gait Real-time Analysis Interactive Lab-based 6-minute walk test

**DOI:** 10.1371/journal.pone.0190099

**Published:** 2017-12-28

**Authors:** Wai-Yan Liu, Martijn A. Spruit, Jeannet M. Delbressine, Paul J. Willems, Frits M. E. Franssen, Emiel F. M. Wouters, Kenneth Meijer

**Affiliations:** 1 Department of Research and Education, CIRO, Horn, the Netherlands; 2 Department of Human Movement Science, NUTRIM School of Nutrition and Translational Research in Metabolism, Maastricht University Medical Centre+, Maastricht, the Netherlands; 3 Department of Respiratory Medicine, NUTRIM School of Nutrition and Translational Research in Metabolism, Maastricht University Medical Centre+, Maastricht, the Netherlands; 4 Department of Respiratory Medicine, Maastricht University Medical Centre+, Maastricht, the Netherlands; Universite de Bretagne Occidentale, FRANCE

## Abstract

**Background and aim:**

Overground gait assessment is limited by the analysis of multiple strides or both spatiotemporal gait characteristics, while fixed speed treadmill walking restricts natural gait speed variations. The Gait Real-time Analysis Interactive Lab (GRAIL)-based 6-minute walk test (6MWT) enables 3D motion analysis and self-paced treadmill walking, and could provide insight in gait alterations in patients with chronic obstructive pulmonary disease (COPD). The aim of this study is to compare spatiotemporal gait characteristics between patients with COPD and healthy elderly during the GRAIL-based 6MWT.

**Materials and methods:**

Eighty COPD patients (60% male; 62±7 years; FEV_1_:56±19% predicted) and 38 healthy elderly (63% male; 62±6 years; FEV_1_:119±17% predicted) performed two GRAIL-based 6MWTs. Mean differences and coefficient of variation of spatiotemporal gait characteristics were calculated using the trial with the largest walk distance. Sub-analyses were conducted to account for walking speed differences between groups, and muscle strength and COPD severity within the patient group.

**Results:**

COPD patients showed increased temporal gait characteristics, decreased stride and step lengths, and increased gait variability compared to healthy elderly (p<0.01). Stride length variability remained increased in COPD after correction for walking speed (MD:0.98%, CI:0.36–1.61, p = 0.003). Reduced quadriceps strength did not translate into altered gait characteristics, while COPD severity is associated with stride time (left MD:-0.02s, CI:-0.04–0.01, p = 0.003; right MD:-0.02s, CI:-0.04–0.01, p = 0.003).

**Discussion:**

COPD patients performed the GRAIL-based 6MWT differently compared to healthy elderly. Further research should use other variability measures to investigate gait characteristics in COPD, to assess subtle alterations in gait and to enable development of rehabilitation strategies to improve gait, and possibly balance and fall risk in COPD. Other lower limb muscle groups should be considered when investigating gait alterations in COPD.

**Conclusion:**

COPD patients have different gait characteristics compared to healthy elderly. Independent of walking speed, COPD patients demonstrate increased stride length variability during the GRAIL-based 6MWT compared to healthy elderly.

## Introduction

Patients with chronic obstructive pulmonary disease (COPD) report walking as one of the most problematic activity in daily life [1]. Patients with COPD walk less in daily life [2] and achieve shorter walk distances during the 6-minute walk test (6MWT) compared to healthy subjects [3]. Walking distance is an important parameter in the evaluation of treatment and as a prognostic value, in which walking distances <350 m on the 6MWT are associated with increased mortality [4]. In addition, patients with COPD demonstrate extrapulmonary manifestations affecting the muscular system, resulting in muscular dysfunction [5, 6] and most probably in gait (walking) alterations.

Gait assessment could provide insight in the biomechanical factors associated with the reduced walk distances in patients with COPD. To date, several studies have explored gait in COPD [3, 7–10]. One study reported that gait alterations, such as limping and shuffling, are associated with disease severity in COPD [11]. Patients with COPD also demonstrate decreased cadence, shorter step lengths, increased time spent in double support and a lack of increase in peak ankle dorsiflexion moment after the onset of breathlessness or leg tiredness compared to healthy subjects, while walking at their comfortable speed [7–9]. Increased balance disturbances in mediolateral direction in patients with COPD were observed during the 6MWT [3]. Furthermore, patients with COPD walk with an larger step time and smaller step width variability during fixed speed treadmill [12].

Most studies recorded gait in patients with COPD during overground walking, using accelerometry [3], a pressure sensitive mat [7], 2D or 3D motion capture systems [8, 9]. However, the latter three methods can only assess a limited number of consecutive strides, due to space and/or equipment constraints. Gait analysis using instrumented treadmills could be an alternative to overground gait analysis, as they require less laboratory space [13]. However, treadmill walking at fixed speeds restricts subjects to walk with speed variations, resulting in less natural stride variability. Self-paced treadmill walking, involving a feedback-regulated treadmill that allows subjects to walk at their preferred speed, is suggested to be a suitable alternative to fixed speed treadmill walking in gait analysis [13]. In addition, accelerometry enables the recording of multiple strides, but cannot reliably capture the spatial gait characteristics [14].

The Gait Real-time Analysis Interactive Lab (GRAIL) enables self-paced treadmill walking combined with 3D motion capture and could overcome the limitations in overground and fixed speed treadmill walking. Previous studies observed similar spatiotemporal, kinetic and kinematic gait characteristics in self-paced and fixed speed treadmill walking using the GRAIL system [13, 15]. Moreover, gait speed in self-paced treadmill was comparable to overground walking when using a similar system as the GRAIL [14]. Spatiotemporal gait characteristics can therefore be assessed accurately in patients with COPD over multiple consecutive strides and during exercise testing, such as during a 6MWT. As patients with COPD are able to perform the 6MWT using the GRAIL with minimal differences compared to the overground 6MWT [16], additional outcomes can be obtained to identify gait alterations within this population. In addition, as the 6MWT is a submaximal exercise test, challenging the body to walk at speeds outside of the comfortable walking speed can reveal declines in gait or associations in gait characteristics that are otherwise camouflaged at their comfortable walking speed [17].

The aim of the present study is to compare spatiotemporal gait characteristics between patients with COPD and healthy elderly during the GRAIL-based 6MWT (with and without adjustment for expected differences in walking speed; and within the patient sample after stratification for quadriceps muscle strength or COPD severity, described as the degree of airflow limitation). It was hypothesized that patients with COPD would demonstrate alterations in their spatiotemporal gait characteristics as compared to healthy elderly.

## Materials and methods

### Study design

This cross-sectional study was conducted in CIRO, a centre of expertise for chronic organ failure in Horn, the Netherlands. Patients with COPD, assessed by spirometry (Carefusion, San Diego, CA, USA) according to the Global Initiative for Chronic Obstructive Lung Disease (GOLD) criteria (post-bronchodilator Forced Expiratory Volume in one second /Forced Vital Capacity; FEV_1_/FVC ratio <0.70), were recruited at regular pre-rehabilitation assessment. Patients with walking aids, chronic oxygen therapy, orthopaedic ailments and/or neuromuscular co-morbidities affecting their walking patterns were excluded, as well as patients with a history of lung cancer, asthma, sarcoidosis, tuberculosis and/or lung surgery. Healthy elderly were recruited amongst healthy subjects who participated in previous trials [18], from co-workers or from healthy relatives of patients. These healthy elderly subjects were included as the non-COPD group (post-bronchodilator FEV_1_/FVC ratio >0.70), if subjects did not have any self-reported neuromuscular and/or orthopaedic ailments. Spirometry and electrocardiography were conducted prior to the GRAIL-based 6MWT. Then, quadriceps muscle strength was measured (Biodex System 4 Pro, Biodex Medical Systems, Inc., New York, USA). Quadriceps muscle strength was calculated as the peak torque of % predicted, based on Borges et al. [19]. Participants performed thirty volitional maximal contractions at an angular velocity of 90 degrees per second. This study (M13-1374) complied with the Declaration of Helsinki and was approved by the Medical research Ethics Committees United (MEC-U) in the Netherlands. This study is registered at the Dutch Trial Register (NTR4421). Written consent was obtained from all participants.

### Assessment of gait

**Fig 1:**
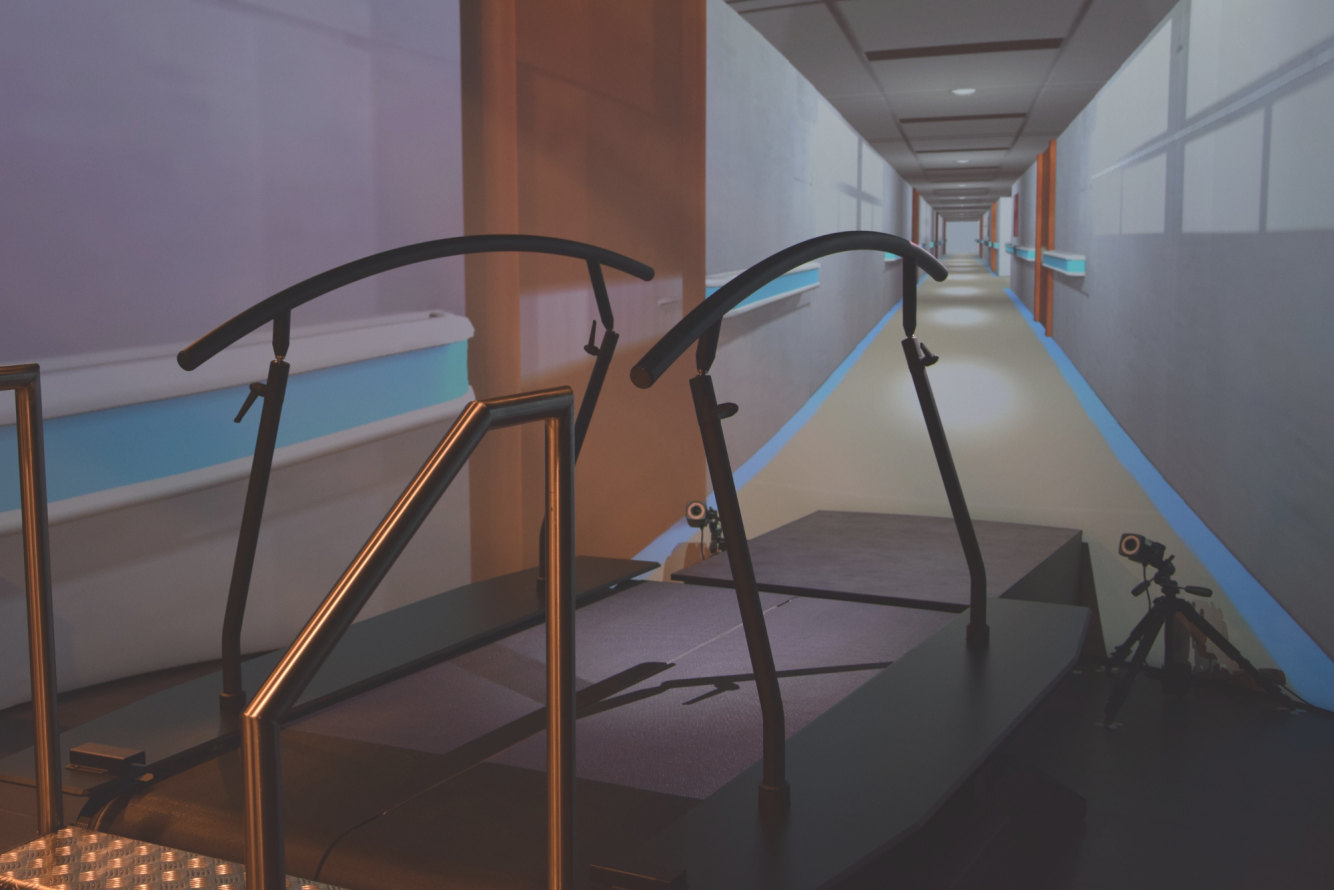
The GRAIL system.

**Fig 2:**
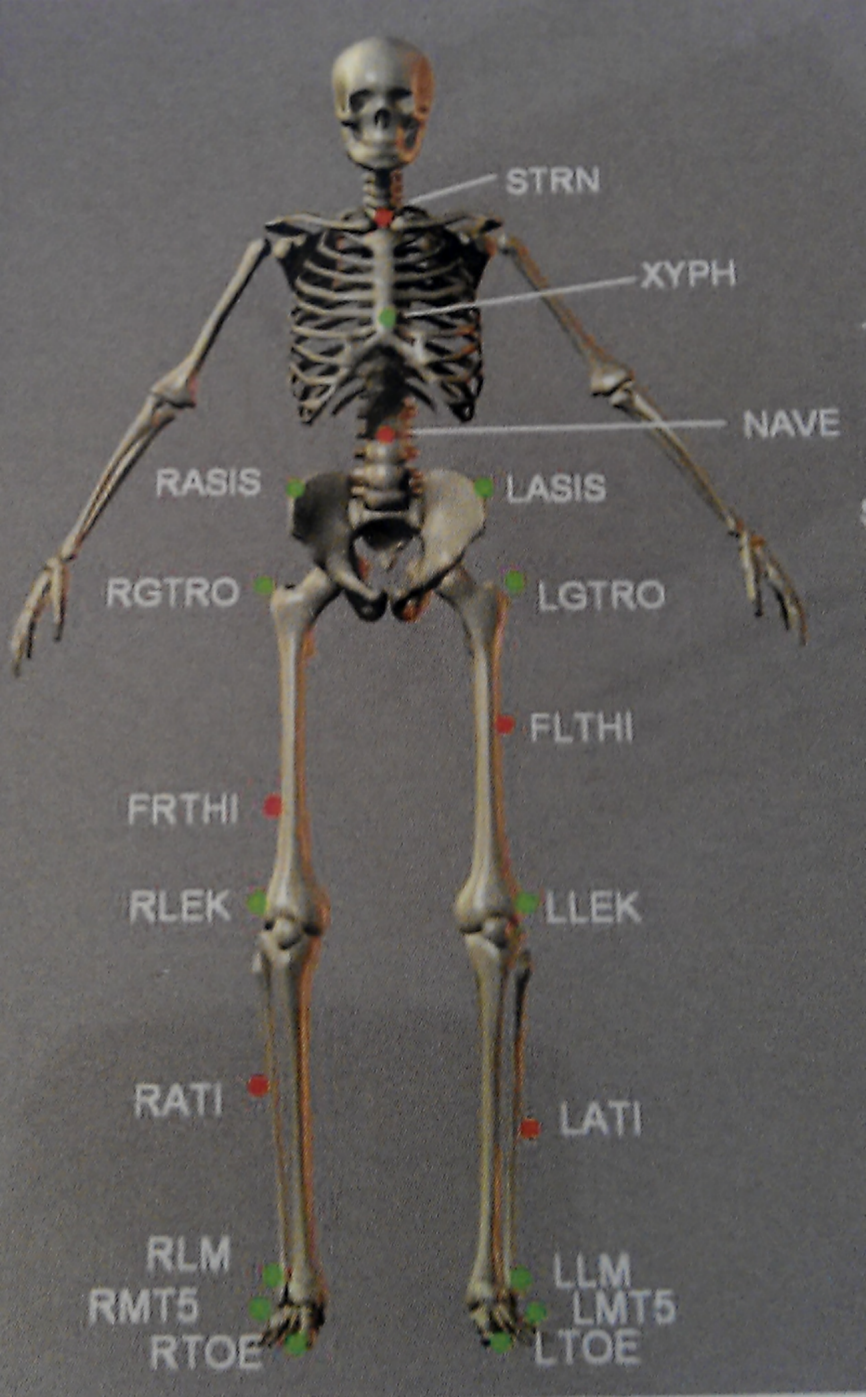
The human body model of the lower limb. Anatomical landmarks are the sternum (STRN), xiphoid (XYPH), navel (NAVE), T10, sacral bone, anterior and posterior superior iliac spine (ASIS and PSIS), greater trochanter of the femur (TROCH), lateral between the greater trochanter and lateral femoral epicondyle (LTHI), lateral epicondyle of the knee (LEK), lateral between the lateral femoral epicondyle and lateral malleolus (ATI), lateral malleoli (LM), heel (HEE), tip of the first metatarsal (TOE) and the fifth metatarsal heads (MT5).

Each participant performed two 6MWT’s using the GRAIL (Motekforce Link, Amsterdam, the Netherlands), a 3D motion capture system with a instrumented dual-belt treadmill and virtual reality on a 180 degrees projection screen (Fig 1). Optical flow of the virtual reality environment was synchronised with the treadmill velocity. Subjects wore tight fitting shorts. Twenty-five reflective markers were placed on anatomical landmarks of each participant according to the Human Body Model (HBM) of the lower limb (Fig 2) [20]. The 3D marker trajectories were collected (100 Hz) with a ten camera 3D motion capture system (Vicon Nexus, Oxford Metrics Ltd., Oxford, UK) and processed in D-flow (Motekforce Link, Amsterdam, the Netherlands). Ground reaction force data from heel contact to toe off were collected using integrated force plates (Forcelink, 12 channels, sample frequency 1000 Hz). Participants were able to self-adjust treadmill speed via a feedback-regulated algorithm in D-flow (Motekforce Link, Amsterdam, the Netherlands). One familiarisation session was performed in order to become accustomed to self-paced treadmill walking and the virtual reality hallway environment. The first GRAIL-based 6MWT was performed after the familiarisation session. Both the familiarisation and the first GRAIL-based 6MWT were conducted during pre-rehabilitation assessment. Patients performed the second GRAIL-based 6MWT between pre-rehabilitation assessment and the first week of pulmonary rehabilitation. Healthy elderly performed two GRAIL-based 6MWTs within one day with at least 45 minutes of rest in between the two tests. Perceived dyspnoea and fatigue were assessed before and after the GRAIL-based 6MWT using a BORG scale. Heart rate and pulse oxygen saturation (SpO2) levels were measured prior and post each GRAIL-based 6MWT using pulse oximetry (Nonin, Care Fusion, San Diego, USA). The instructions of the GRAIL-based 6MWT were provided according to the European Respiratory Society/American Thoracic Society guidelines [21].

**Fig 3:**
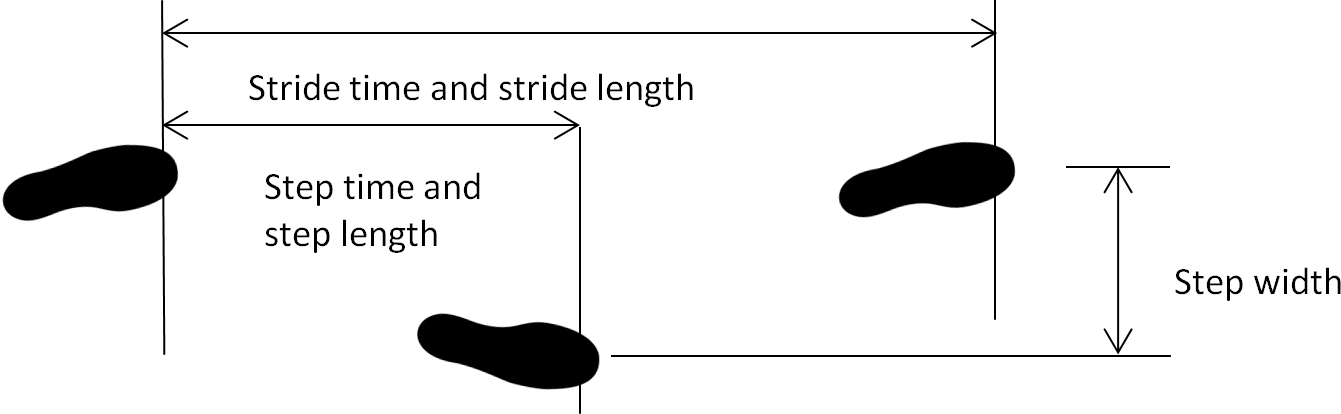
Spatiotemporal gait characteristics.

The GRAIL-based 6MWT with the longest walk distance for each subject was used for data processing and analysis. The first 60 seconds of the data were excluded to minimize start-up effects and 15 seconds prior to the end of the test were excluded to minimize deceleration of the treadmill speed towards the end of the test (S1 Appendix). All steps were included for analysis. Gait parameters were determined using a custom program in Matlab (MathWorks Inc., Natick, USA. S2 Appendix). We computed the following spatiotemporal gait characteristics: walking speed (m/s), cadence (steps/min), double support time (s), stride time (s), stride length (m) and step width (m). Step time (s), stance time (s), swing time (s) and step length (m) were computed for left and right separately. Double support time was calculated as the duration of both feet on the force plates. Stride time was calculated as the time from one heel contact to the next ipsilateral heel contact. Stride length was defined as the distance between the toe marker and the ipsilateral toe marker at each heel contact in the anterior-posterior direction. Step width was defined as the distance between the toe marker in mediolateral direction between both feet at heel strike. Step time was calculated as the time from one heel contact to the contralateral heel contact. Stance time was calculated as the duration between heel strike and toe off of the ipsilateral leg. Swing time was calculated as the duration between toe of and heel strike of the ipsilateral leg. Step length was defined as the distance between the toe marker in anteroposterior direction between both feet at heel strike (Fig 3).

To assess the magnitude of variability in spatiotemporal gait characteristics, coefficient of variation ([standard deviation of the gait characteristic divided by the mean] x 100%) was calculated for double support time, stride time, stride length, step width, step time, stance time, swing time and step length. In addition, we included commonly reported parameters of the 6MWT, such as 6MWD, mean walking speed, BORG dyspnoea score, BORG fatigue score, SpO2 level and heart rate.

### Statistical analyses

Sample size and power sample size calculations were based on outcomes of Annegarn et al. [3] using the inter-stride trunk acceleration variability of patients with COPD and healthy elderly. Thirty-five participants in each group would provide 90% power at alpha 0.05 (two-tailed) to detect differences between patients with COPD and healthy elderly (63.2±14.0% and 73.7±12.5%, respectively). As spatiotemporal gait characteristics during the GRAIL-based 6MWT have not been assessed, larger sample sizes were used for analyses.

All variables were checked for outliers (S3 Appendix) and normality distribution using the Shapiro Wilk test. Differences between patients with COPD and healthy elderly were studied using independent samples T-tests and non-parametric independent T-tests. A posteriori, the first sub-analysis of 14 patients and 14 healthy elderly with comparable walking distances was performed to assess the differences between the groups independent of walking speed. To assess possible influences of muscular dysfunction on gait, a second sub-analysis was conducted to assess differences in spatiotemporal gait characteristics between patients with low (isokinetic peak torque <70% predicted) and high (isokinetic peak torque >70% predicted) quadriceps muscle strength in COPD. This value corresponded to the cut-off value of 2 standard deviations below the mean isokinetic peak torque for males and females in age group 60 years [19]. In the third sub-analysis, we compared spatiotemporal gait characteristics between patients with mild to moderate COPD (GOLD I-II) and severe to very severe COPD (GOLD III-IV). Mean values, mean differences (MD), standard deviations, coefficients of variation and confidence intervals (CI) were calculated. A p-value below the conventional level of significance (p<0.01) was considered statistically significant. Statistical analyses were performed using SPSS, version 22.0 (IBM, New York, USA).

## Results

### Demographics

A sample of 80 patients and 38 healthy elderly were analysed for this study. Baseline characteristics of the study population are presented in Table 1. Majority of the patients had moderate to severe COPD.

**Table 1:** Demographics of subjects.

Characteristics		Patients with COPD	Healthy elderly	p-value
Subjects, n		80	38	
Age, years		62.3 ± 7.2	62.1 ± 6.3	0.718
Males, n		48 (60.0)	24 (63.2)	
Weight, kg		75.9 ± 16.9^†^	79.3 ± 13.0	0.273
Height, m		1.70 ± 0.09	1.72 ± 0.08^†^	0.182
BMI, kg/m^2^		26.3 ± 5.1	26.8 ± 3.1	0.444
FEV_1_/FVC		0.41 ± 0.11	0.77 ± 0.05^†^	<0.001*
FEV_1_% predicted		55.8 ± 19.4	118.6 ± 16.5^†^	<0.001*
Never smoker, n		1 (1.3)	14 (36.8)	
Former smoker, n		63 (78.8)	23 (60.5)	
Current smoker, n		6 (7.5)	1 (2.6)	
GOLD group, n	I	10 (12.5)		
	II	35 (43.8)		
	III	29 (36.3)		
	IV	6 (7.5)		

Data are presented as mean ± standard deviation or n (%), unless otherwise stated. COPD: chronic obstructive pulmonary disease; BMI: body mass index; FEV_1_: forced expiratory volume in 1 s; FVC: forced vital capacity; GOLD: Global Initiative for Chronic Obstructive Lung Disease.

*: indicates a significant (p<0.01) difference between patients and healthy elderly.

^†^: indicates a non-normal distributed variable.

### The GRAIL-based 6MWT and spatiotemporal gait characteristics

Patients with COPD achieved on average a shorter 6MWD compared to healthy elderly (494±80m and 689±64m, p<0.001, respectively, Table 2). Oxygen saturation level decreased significantly during the GRAIL-based 6MWT in patients (-2.7±4.3%, p<0.001) and exercise induced oxygen desaturation was found in 20% of the patients (SpO_2_nadir ≤ 88%). Patients with COPD showed less increase in heart rate and experienced more dyspnoea and fatigue after the GRAIL-based 6MWT compared to healthy elderly.

**Table 2:** Best GRAIL-based 6MWT outcomes in patients with COPD and healthy elderly.

	Patients with COPD n = 80	Healthy elderly n = 38	p-value
6MWD, m	493.5 ± 79.7	689.3 ± 64.3	<0.001*
Walking speed, m/s	1.4 ± 0.2	1.9 ± 0.2	<0.001*
Pre SpO_2_, %	95.1 ± 1.5^†^	97.1 ± 1.0^†^	<0.001*
Post SpO_2_, %	92.3 ± 4.6^#^^†^	97.1 ± 1.2^†^	<0.001*
Pre HR, bpm	82.0 ± 13.9	65.9 ± 11.9	<0.001*
Post HR, bpm	103.0 ± 18.1^#^	98.6 ± 22.7^#^	0.295
Pre Dyspnoea, score	1.2 ± 1.2^†^	0.2 ± 0.3^†^	<0.001*
Post Dyspnoea, score	4.8 ± 2.3^#^^†^	1.1 ± 1.0^#^^†^	<0.001*
Pre Fatigue, score	1.3 ± 1.3^†^	0.3 ± 0.4^†^	<0.001*
Post Fatigue, score	4.5 ± 2.3^#^^†^	1.3 ± 1.1^#^^†^	<0.001*

Data are presented as mean ± standard deviation. COPD: chronic obstructive pulmonary disease. 6MWD: 6-minute walk distance; SpO_2_: peripheral capillary oxygen saturation; HR: heart rate; bpm: beats per minute.

*: indicates a significant (p<0.01) difference between patients with COPD and healthy elderly.

^#^: indicates a significant (p<0.01) difference between pre and post values.

^†^: indicates a non-normal distributed variable.

Patients with COPD demonstrated decreased cadence compared to healthy elderly (MD: -17.36steps/min, CI:-21.10- -13.61, p<0.001, Table 3). Mean duration of temporal gait characteristics was significantly longer in patients with COPD, while stride length was shorter. No differences were found between left and right spatiotemporal gait characteristics. Patients show increased variability in double support time (MD: 1.18%, CI: 0.69–1.66, p = 0.001), stride length (MD: 1.82%, CI: 1.39–2.25, p<0.001), stance time (left MD: 0.58%, CI: 0.29–0.87, p = 0.002; right MD: 0.57%, CI: 0.28–0.86, p = 0.003), and step length (left MD: 1.77%, CI: 1.32–2.22, p<0.001; right MD: 1.74%, CI:1.29–2.19, p<0.001). Step width variability did not reach a statistical significant difference between the groups (MD: -2.98%, CI: -5.39- -0.57, p = 0.011). The distribution of spatiotemporal gait characteristics for both groups are presented in S4 Appendix.

**Table 3:** Spatiotemporal gait characteristics in patients with COPD and healthy elderly.

		Patients with COPD n = 80	Healthy elderly n = 38	p-value
**Mean values**				
	Cadence, steps/min	118.6 ± 10.3	136.0 ± 8.0	<0.001*
	Double support time, s	0.28 ± 0.04	0.24 ± 0.02	<0.001*
	Stride time, s	1.02 ± 0.09	0.89 ± 0.05	<0.001*
	Stride length, m	1.43 ± 0.18	1.73 ± 0.14	<0.001*
	Step width, m	0.18 ± 0.04	0.17 ± 0.05	0.056
**Left**	Step time, s	0.51 ± 0.04	0.44 ± 0.03	<0.001*
	Stance time, s	0.65 ± 0.06	0.56 ± 0.03	<0.001*
	Swing time, s	0.37 ± 0.04	0.33 ± 0.02	<0.001*
	Step length, m	0.71 ± 0.09	0.86 ± 0.07	<0.001*
**Right**	Step time, s	0.51 ± 0.05	0.44 ± 0.03	<0.001*
	Stance time, s	0.65 ± 0.06	0.56 ± 0.03	<0.001*
	Swing time, s	0.37 ± 0.04	0.33 ± 0.02	<0.001*
	Step length, m	0.72 ± 0.10	0.87 ± 0.07	<0.001*
**Coefficient of variation**				
	Cadence, %	2.99 ± 1.26^†^	2.65 ± 0.73^†^	0.154
	Double support time, %	6.37 ± 1.90^†^	5.19 ± 0.77	0.001*
	Stride time, %	1.97 ± 0.77^†^	1.64 ± 0.32	0.102
	Stride length, %	3.87 ± 1.78^†^	2.03 ± 0.54^†^	<0.001*
	Step width, %	13.97 ± 5.35^†^	16.95 ± 7.66^†^	0.011
**Left**	Step time, %	2.54 ± 0.82^†^	2.23 ± 0.46^†^	0.087
	Stance time, %	2.94 ± 1.05^†^	2.36 ± 0.54^†^	0.002*
	Swing time, %	2.36 ± 0.56^†^	2.70 ± 0.46^†^	0.580
	Step length, %	4.35 ± 1.85^†^	2.58 ± 0.58	<0.001*
**Right**	Step time, %	2.58 ± 0.82^†^	2.26 ± 0.50^†^	0.081
	Stance time, %	2.91 ± 1.06^†^	2.34 ± 0.53	0.003*
	Swing time, %	2.41 ± 0.65^†^	2.28 ± 0.47^†^	0.561
	Step length, %	4.35 ± 1.82^†^	2.61 ± 0.61^†^	<0.001*

Data are presented as mean ± standard deviation.

*: significant difference between patients with COPD and healthy elderly (p<0.01).

^†^: indicates a non-normal distributed variable.

### Sub-analyses

Sub-analysis of 14 patients and 14 healthy elderly subjects with comparable walking distances was performed to assess differences between the groups independent of walking speed (MD: -10.2m, CI: -36.7–16.3, p = 0.437, Table 4). Heart rate, dyspnoea and fatigue scores significantly differed in pre and post exercise testing within each group and between the groups. Mean spatiotemporal gait characteristics in patients with COPD did not differ with healthy elderly. Variability in gait characteristics showed differences in stride length. (MD: 0.98%, CI: 0.35–1.61, p = 0.003) and partly in step length (left MD: 1.07%, CI: 0.36–1.78, p = 0.005; right MD: 1.10%, CI: 0.23–1.97, p = 0.014) between patients and healthy subjects (Table 5). No asymmetry was found between left and right spatiotemporal gait characteristics. Distribution of spatiotemporal gait characteristics for both groups are presented in S5 Appendix.

**Table 4:** Sub-analysis of the GRAIL-based 6MWT outcomes in groups with comparable walking speeds.

	Patients with COPD n = 14	Healthy elderly n = 14	p-value
FEV_1_/FVC	44.3 ± 10.1	77.7 ± 5.8	<0.001
FEV_1_% predicted	64.2 ± 18.2	116.7 ± 23.1	<0.001
6MWD, m	611.8 ± 30.2	622.0 ± 37.7	0.437
Walking speed, m/s	1.7 ± 0.1^†^	1.7 ± 0.1	0.701
Pre SpO_2_, %	95.8 ± 1.7	97.4 ± 0.6^†^	0.003*
Post SpO_2_, %	94.2 ± 3.7^†^	97.0 ± 1.0^†^	0.015
Pre HR, bpm	78.7 ± 14.8	65.6 ± 9.9	0.010
Post HR, bpm	108.8 ± 19.1^#^	92.4 ± 18.3^#^	0.028
Pre Dyspnoea, score	1.1 ± 0.9^†^	0.2 ± 0.3^†^	0.001*
Post Dyspnoea, score	5.0 ± 2.2^#^^†^	0.8 ± 0.5^#^^†^	<0.001*
Pre Fatigue, score	0.9 ± 0.9^†^	0.3 ± 0.5^†^	0.006*
Post Fatigue, score	4.8 ± 2.4^#^^†^	0.9 ± 0.9^#^^†^	<0.001*

Data are presented as mean ± standard deviation. COPD: chronic obstructive pulmonary disease. 6MWD: 6-minute walk distance; SpO_2_: peripheral capillary oxygen saturation; HR: heart rate; bpm: beats per minute.

*: indicates a significant (p<0.01) difference between patients and healthy elderly.

^#^: indicates a significant (p<0.01) difference between pre and post values.

^†^: indicates a non-normal distributed variable.

**Table 5:** Sub-analysis of the spatiotemporal gait characteristics in groups with comparable walking speeds.

		Patients with COPD n = 14	Healthy elderly n = 14	p-value
**Mean values**				
	Cadence, steps/min	128.40 ± 12.00	132.00 ± 4.86	0.306
	Double support time, s	0.25 ± 0.02^†^	0.24 ± 0.02	0.227
	Stride time, s	0.94 ± 0.08	0.91 ± 0.03	0.212
	Stride length, m	1.62 ± 0.17	1.59 ± 0.07	0.552
	Step width, m	0.18 ± 0.04	0.16 ± 0.05^†^	0.260
**Left**	Step time, s	0.47 ± 0.04	0.46 ± 0.02	0.209
	Stance time, s	0.59 ± 0.04	0.57 ± 0.03	0.158
	Swing time, s	0.35 ± 0.04	0.34 ± 0.02	0.350
	Step length, m	0.82 ± 0.09	0.80 ± 0.04	0.512
**Right**	Step time, s	0.47 ± 0.04	0.45 ± 0.02	0.213
	Stance time, s	0.60 ± 0.05	0.58 ± 0.03	0.180
	Swing time, s	0.34 ± 0.04	0.33 ± 0.02	0.281
	Step length, m	0.82 ± 0.08	0.80 ± 0.04	0.422
**Coefficient of variation**				
	Cadence, %	2.81 ± 0.84	2.44 ± 0.87^†^	0.104
	Double support time, %	5.81 ± 1.36	4.74 ± 0.75	0.015
	Stride time, %	1.74 ± 0.48	1.45 ± 0.27	0.067
	Stride length, %	3.03 ± 0.98	2.04 ± 0.59	0.003*
	Step width, %	16.17 ± 5.92^†^	15.09 ± 4.82	0.963
**Left**	Step time, %	2.41 ± 0.81^†^	1.93 ± 0.27	0.178
	Stance time, %	2.64 ± 0.90^†^	2.09 ± 0.34	0.050
	Swing time, %	2.35 ± 0.70^†^	2.02 ± 0.28	0.352
	Step length, %	3.61 ± 1.12	2.54 ± 0.63	0.005*
**Right**	Step time, %	2.43 ± 0.85^†^	2.05 ± 0.40	0.265
	Stance time, %	2.62 ± 0.89^†^	2.02 ± 0.33	0.062
	Swing time, %	2.35 ± 0.73^†^	2.10 ± 0.33	0.839
	Step length, %	3.72 ± 1.40^†^	2.62 ± 0.67	0.014

Data are presented as mean ± standard deviation. COPD: chronic obstructive pulmonary disease.

*: indicates a significant (p<0.01) difference between patients and healthy elderly.

^†^: indicates a non-normal distributed variable.

In the second sub-analysis, the influence of muscular dysfunction on gait characteristics was assessed within the COPD group. Female COPD subjects were less represented in the high quadriceps muscle strength group (n = 8) compared to male COPD subjects in the high muscle strength group (n = 25). Therefore, comparisons between quadriceps muscle strength were conducted for males and females separately (Table 6). Difference in muscle strength in male subjects was -23.64% predicted (CI: -29.7- -17.6, p<0.001) and -29.7% predicted (CI: -35.5 - -24.0, p<0.001) in female subjects. No significant differences in mean spatiotemporal gait characteristics were found between low and high quadriceps muscle strength groups within male and female subjects.

**Table 6:** Spatiotemporal gait characteristics of patients within low and high muscle strength group.

			Male			Female		
			Low muscle strength group n = 18	High muscle strength group n = 25	p-value	Low muscle strength group n = 18	High muscle strength group n = 8	p-value
**Mean values**								
	FEV_1_/FVC		37.92 ± 10.57	42.95 ± 12.06	0.163	41.29 ± 12.27	`44.25 ± 11.91	0.549
	FEV_1_% predicted		51.12 ± 15.66	58.59 ± 19.60	0.189	56.27 ± 22.88	53.80 ± 14.94	0.783
	Isokinetic strength, Nm		102.57 ± 18.71	144.09 ± 23.86	<0.001*	66.72 ± 8.00	97.60 ± 12.11	<0.001^#^
	Isokinetic strength % predicted		60.39 ± 9.38^†^	84.03 ± 9.86^†^	<0.001*	56.82 ± 5.92	86.57 ± 7.88	<0.001^#^
	6MWD, m		484.61 ± 73.87	518.75 ± 84.64	0.177	488.61 ± 87.00	481.33 ± 79.03	0.841
	Walking speed, m/s		1.38 ± 0.21	1.49 ± 0.24	0.117	1.40 ± 0.25	1.38 ± 0.24	0.832
	Cadence, steps/min		113.60 ± 9.30	118.39 ± 11.07^†^	0.143	123.01 ± 9.55	119.47 ± 6.43	0.351
	Double support time, s		0.29 ± 0.03	0.29 ± 0.04^†^	0.695	0.26 ± 0.05	0.29 ± 0.04	0.170
	Stride time, s		1.06 ± 0.09	1.02 ± 0.09	0.133	0.98 ± 0.08	1.01 ± 0.06	0.426
	Stride length, m		1.45 ± 0.18	1.50 ± 0.18	0.330	1.36 ± 0.18	1.38 ± 0.21	0.803
	Step width, m		0.19 ± 0.05	0.20 ± 0.04	0.472	0.15 ± 0.03	0.18 ± 0.03	0.056
**Left**	Step time, s		0.54 ± 0.05	0.51 ± 0.04^†^	0.108	0.50 ± 0.04	0.51 ± 0.03	0.549
	Stance time, s		0.67 ± 0.05^†^	0.65 ± 0.06	0.220	0.62 ± 0.06^†^	0.65 ± 0.04	0.149
	Swing time, s		0.39 ± 0.04	0.37 ± 0.03^†^	0.083	0.36 ± 0.03	0.36 ± 0.02^†^	0.617
	Step length, m		0.73 ± 0.09	0.74 ± 0.11	0.749	0.69 ± 0.09	0.69 ± 0.11	0.992
**Right**	Step time, s		0.53 ± 0.05	0.51 ± 0.05	0.175	0.49 ± 0.04	0.50 ± 0.03	0.358
	Stance time, s		0.68 ± 0.05^†^	0.66 ± 0.06	0.159	0.63 ± 0.06^†^	0.65 ± 0.04	0.201
	Swing time, s		0.38 ± 0.04	0.37 ± 0.03^†^	0.141	0.36 ± 0.03	0.36 ± 0.02	0.926
	Step length, m		0.72 ± 0.09	0.77 ± 0.10^†^	0.046	0.68 ± 0.10	0.70 ± 0.10	0.634

Data are presented as mean ± standard deviation.

*: indicates a significant (p<0.01) difference between males in low and high muscle strength.

^#^: indicates a significant (p<0.01) difference between females in low and high muscle strength.

^†^: indicates a non-normal distributed variable.

In the third sub-analysis, the influence of the degree of airflow limitation on gait characteristics was assessed within the COPD group. Spatiotemporal gait characteristics were compared between patients with mild-moderate COPD (68.9 ± 1.9% predicted) and severe-very severe COPD (38.0 ± 8.0% predicted; Table 7). Mean swing time was significantly different between the groups (left MD: -0.02s, CI: -0.04- -0.01, p = 0.003; right MD: -0.02s, CI:-0.04–0.01, p = 0.003).

**Table 7:** Spatiotemporal gait characteristics of patients within mild-moderate and severe-very severe COPD.

		Mild-moderate COPD n = 45	Severe-very severe COPD n = 35	p-value
**Mean values**				
	FEV_1_/FVC	47.98 ± 8.55	32.98 ± 8.86^†^	<0.001^*^
	FEV_1_% predicted	69.70 ± 13.24	37.98 ± 8.04	<0.001^*^
	6MWD, m	507.05 ± 87.98	480.55 ± 65.70	0.141
	Walking speed, m/s	1.45 ± 0.25	1.37 ± 0.18	0.093
	Cadence, steps/min	120.54 ± 10.84	116.12 ± 9.00	0.055
	Double support time, s	0.29 ± 0.03	0.28 ± 0.04	0.234
	Stride time, s	1.00 ± 0.09	1.04 ± 0.08	0.048
	Stride length, m	1.44 ± 0.21	1.41 ± 0.14	0.534
	Step width, m	0.19 ± 0.04	0.17 ± 0.04^†^	0.043
**Left**	Step time, s	0.51 ± 0.05	0.52 ± 0.04	0.088
	Stance time, s	0.64 ± 0.06	0.66 ± 0.06	0.315
	Swing time, s	0.36 ± 0.04^†^	0.38 ± 0.03	0.002^*^
	Step length, m	0.72 ± 0.10	0.70 ± 0.08	0.332
**Right**	Step time, s	0.50 ± 0.05	0.52 ± 0.04	0.055
	Stance time, s	0.65 ± 0.06	0.66 ± 0.06	0.313
	Swing time, s	0.36 ± 0.04	0.38 ± 0.03	0.003^*^
	Step length, m	0.72 ± 0.11	0.71 ± 0.08^†^	0.186

Data are presented as mean ± standard deviation.

*: indicates a significant (p<0.01) difference between mild-moderate COPD and severe-very severe COPD.

^†^: indicates a non-normal distributed variable.

## Discussion

This study demonstrates that patients with COPD have different spatiotemporal gait characteristics as compared to healthy elderly during a self-paced treadmill based 6MWT. Patients achieved shorter walk distances with a lower cadence and increased duration of temporal gait characteristics. Patients took shorter steps and showed increased variability in double support time, stride length, stance time and step length. Sub-analysis showed that differences in mean spatiotemporal gait characteristics are mainly attributed to differences in walking speed between the groups. However, variability in stride length remained higher in patients with COPD compared to healthy elderly in the sub-analysis of subject groups with comparable walking speeds. Stratifying patients by quadriceps muscle strength did not distinguish differences in spatiotemporal gait characteristics. Patients with mild-moderate COPD showed decreased swing time as compared to severe-very severe COPD.

This is the first study to investigate spatiotemporal gait characteristics during a self-paced treadmill based 6MWT in patients with COPD using the GRAIL. Walking speed and cadence were decreased in patients with COPD, which is in line with previous studies [3, 22, 23]. Reduced gait speed and cadence could reflect respiratory limitations associated with muscular dysfunction in COPD [22, 24, 25]. These gait alterations might be an adaptation mechanism to cope with impaired walking endurance due to the lack of oxygen supply and lower limb muscle weakness. Taking slower steps decreases the oxygen demands of the leg muscles and thus allows for increased walking distance in patients with impaired lung function [26]. Increased step width could be an adaptation to increase the base of support during the double support phase of gait [9]. However, our results did not support differences in step width between patients with COPD and healthy elderly.

Increased variability in double support time, stride length, stance time and step length were found in patients with COPD as compared to healthy elderly. These findings show that patients are less consistent in their walking pattern during the GRAIL-based 6MWT. Step length and stance time variability are increased in older adult fallers as compared to older adult non-fallers [27, 28]. Increased gait variability in COPD could therefore be associated with an increased fall risk within this population. Decreased step width variability in patients with COPD as compared to healthy elderly subjects did not reach a statistical significant difference. However, Yentes et al. [12] suggested that reduced variability in step width may be related to an increased likelihood to a crossover gait. This might indicate that patients are less able to maintain stability, thus predisposing an individual to a fall [29, 30].

Variability in spatiotemporal gait characteristics was calculated as the coefficient of variation, describing the magnitude of variation in each spatiotemporal gait characteristic. When examining the individual variations per gait characteristic, differences between the two groups are observed. However, these differences are cancelled out when comparing the mean values in a subgroup with similar walking speeds. Therefore, other measures that do take the temporal structure of variation into account could provide additional insight into the control of the system while walking [31–34]. Future research might investigate temporal fluctuations of gait characteristics in patients with COPD, to determine subtle alterations in gait in patients with COPD and its associations with fall risk.

The first sub-analysis showed increased variability in stride length in patients with COPD compared to healthy elderly, which indicates that patients with COPD are more variable in their stride lengths irrespective of walking speed differences. Patients with COPD seem to display a larger distribution of variability in their gait characteristics as compared to healthy elderly when walking at similar walking speeds. These patients experienced greater elevated heart rates and report higher dyspnoea and fatigue scores for both pre and post values compared to healthy elderly. These findings direct to the systemic consequences of COPD, resulting in muscular dysfunction and deconditioning [5, 35], in which pulmonary rehabilitation has proven to enhance exercise performance in patients with COPD [36]. Patients with COPD are more susceptible to fatigue [37], possibly explaining the increased fatigue scores in our patients as compared to healthy elderly. Gait alterations in patients with COPD could result in decreased exercise capacity and increased symptoms after the 6MWT. Therefore, gait training could be incorporated within pulmonary rehabilitation programs to improve walking ability and symptoms after exercise testing in COPD.

Previous literature reported correlations between the severity of the disease and functional exercise capacity [38, 39]. However, the current study does not provide evidence for influences of COPD severity on spatiotemporal gait characteristics. Airflow limitation might not have translated to alterations in mean spatiotemporal gait characteristics in COPD. This could be explained by that patients were mostly diagnosed as GOLD II (moderate) and III (severe), whereas patients with GOLD I (mild) and IV (very severe) were less represented in the disease severity groups. Skeletal muscle dysfunction, in particular the quadriceps, is well recognized in COPD and is associated with impaired functional capacity, such as the 6MWT [40, 41]. Our results however did not support differences in spatiotemporal gait characteristics between patients with low and high quadriceps muscle strength. A possible explanation could be that reduced quadriceps muscle strength is not directly related to impaired spatiotemporal gait characteristics in COPD. Alternatively, more distal located lower limb muscles might be more appropriate to be associated with gait characteristics. Previous studies have demonstrated the importance of the triceps surae for propulsion during gait [42–45]. Weakness in ankle dorsiflexors and plantarflexors has been reported to be greater than weakness present in the quadriceps of patients with COPD [46, 47]. In addition, patients with COPD demonstrate a lack of increase in peak ankle dorsiflexion moment after a fatiguing treadmill protocol [9]. Patients with COPD might therefore experience problems in lowering their forefoot after heel strike. Future studies may examine the association between decreased muscle strength in other muscle groups in the lower limbs and altered gait characteristics.

The current study has some limitations. First, subjects walked on a motorized treadmill, which may impede natural walking pattern and its variability. However, previous studies have reported equivocal findings [48–50]. Self-paced treadmill walking was used to provide a more natural control of the walking speed [14, 51], while virtual reality environment could have created a more realistic environment due to the use of a virtual environment enabling optic flow [15]. In addition, self-paced treadmill walking has been validated for healthy subjects during comfortable walking speed [14, 51]. Second, the sample size of female patients in the high quadriceps muscle strength group is small. Consequently, these subjects might not be a comprehensive representation of female patients within the high muscle strength group.

In conclusion, patients with COPD have different spatiotemporal gait characteristics during a self-paced treadmill 6MWT compared to healthy elderly as assessed by the GRAIL. Independent of walking speed, patients with COPD show increased stride length variability during the GRAIL-based 6MWT. Reduced quadriceps strength does not translate into altered spatiotemporal gait characteristics, while COPD severity has impact on swing time. Further research should investigate gait characteristics in patients with COPD using other variability measures, to assess subtle alterations in gait and to enable development of rehabilitation strategies to improve gait, and possibly balance and fall risk in these patients. In addition, other muscle groups in the lower limbs should be taken into account when investigating gait alterations in COPD.

## Supporting information

S1 Appendix Data processing. (PDF)

S2 Appendix Data correction and event detection. (PDF)

S3 Appendix Heterogeneity in COPD and healthy elderly group. (PDF)

S4 Appendix Boxplots of spatiotemporal gait characteristics in the total sample. (PDF)

S5 Appendix Boxplots of spatiotemporal gait characteristics in the sub-analysis. (PDF)

S6 Appendix Dataset. (XLSX)

## References

[1] Annegarn J, Meijer K, Passos VL, Stute K, Wiechert J, Savelberg HH, et al Problematic activities of daily life are weakly associated with clinical characteristics in COPD. Journal of the American Medical Directors Association. 2012;13(3):284–90. Epub 2011/04/01. doi: 10.1016/j.jamda.2011.01.002 . 21450242 10.1016/j.jamda.2011.01.002

[2] Pitta F, Troosters T, Spruit MA, Probst VS, Decramer M, Gosselink R. Characteristics of physical activities in daily life in chronic obstructive pulmonary disease. American journal of respiratory and critical care medicine. 2005;171(9):972–7. Epub 2005/01/25. doi: 10.1164/rccm.200407-855OC . 15665324 10.1164/rccm.200407-855OC

[3] Annegarn J, Spruit MA, Savelberg HH, Willems PJ, van de Bool C, Schols AM, et al Differences in walking pattern during 6-min walk test between patients with COPD and healthy subjects. PloS one. 2012;7(5):e37329 Epub 2012/05/25. doi: 10.1371/journal.pone.0037329 ; PubMed Central PMCID: PMC3356256. 22624017 10.1371/journal.pone.0037329 PMC3356256

[4] Spruit MA, Polkey MI, Celli B, Edwards LD, Watkins ML, Pinto-Plata V, et al Predicting outcomes from 6-minute walk distance in chronic obstructive pulmonary disease. Journal of the American Medical Directors Association. 2012;13(3):291–7. doi: 10.1016/j.jamda.2011.06.009 . 21778120 10.1016/j.jamda.2011.06.009

[5] Gosker HR, van Mameren H, van Dijk PJ, Engelen MP, van der Vusse GJ, Wouters EF, et al Skeletal muscle fibre-type shifting and metabolic profile in patients with chronic obstructive pulmonary disease. The European respiratory journal: official journal of the European Society for Clinical Respiratory Physiology. 2002;19(4):617–25. . 11998989 10.1183/09031936.02.00762001

[6] Wouters EF. Chronic obstructive pulmonary disease. 5: systemic effects of COPD. Thorax. 2002;57(12):1067–70. doi: 10.1136/thorax.57.12.1067 ; PubMed Central PMCID: PMC1758796. 12454303 10.1136/thorax.57.12.1067 PMC1758796

[7] Lahousse L, Verlinden VJ, van der Geest JN, Joos GF, Hofman A, Stricker BH, et al Gait patterns in COPD: the Rotterdam Study. The European respiratory journal: official journal of the European Society for Clinical Respiratory Physiology. 2015;46(1):88–95. doi: 10.1183/09031936.00213214 . 25700390 10.1183/09031936.00213214

[8] Nantsupawat N, Lane P, Siangpraipunt O, Gadwala S, Nugent K. Gait Characteristics in Patients With Chronic Obstructive Pulmonary Disease. Journal of primary care & community health. 2015;6(4):222–6. doi: 10.1177/2150131915577207 . 25801202 10.1177/2150131915577207

[9] Yentes JM, Schmid KK, Blanke D, Romberger DJ, Rennard SI, Stergiou N. Gait mechanics in patients with chronic obstructive pulmonary disease. Respiratory research. 2015;16:31 doi: 10.1186/s12931-015-0187-5 ; PubMed Central PMCID: PMC4351940. 25849481 10.1186/s12931-015-0187-5 PMC4351940

[10] Yentes JM, Rennard SI, Schmid KK, Blanke D, Stergiou N. Patients with COPD Walk with Altered Step Time and Step Width Variability as Compared to Healthy Controls. Annals of the American Thoracic Society. 2017 doi: 10.1513/AnnalsATS.201607-547OC . 28267374 10.1513/AnnalsATS.201607-547OC PMC5566305

[11] Yentes JM, Sayles H, Meza J, Mannino DM, Rennard SI, Stergiou N. Walking abnormalities are associated with COPD: An investigation of the NHANES III dataset. Respiratory medicine. 2011;105(1):80–7. Epub 2010/07/10. doi: 10.1016/j.rmed.2010.06.007 . 20615681 10.1016/j.rmed.2010.06.007

[12] Yentes JM, Rennard SI, Schmid KK, Blanke D, Stergiou N. Patients with Chronic Obstructive Pulmonary Disease Walk with Altered Step Time and Step Width Variability as Compared with Healthy Control Subjects. Annals of the American Thoracic Society. 2017;14(6):858–66. doi: 10.1513/AnnalsATS.201607-547OC . 28267374 10.1513/AnnalsATS.201607-547OC PMC5566305

[13] Sloot LH, van der Krogt MM, Harlaar J. Self-paced versus fixed speed treadmill walking. Gait & posture. 2014;39(1):478–84. doi: 10.1016/j.gaitpost.2013.08.022 . 24055003 10.1016/j.gaitpost.2013.08.022

[14] Plotnik M, Azrad T, Bondi M, Bahat Y, Gimmon Y, Zeilig G, et al Self-selected gait speed—over ground versus self-paced treadmill walking, a solution for a paradox. Journal of neuroengineering and rehabilitation. 2015;12:20 doi: 10.1186/s12984-015-0002-z ; PubMed Central PMCID: PMC4374285. 25881130 10.1186/s12984-015-0002-z PMC4374285

[15] Sloot LH, van der Krogt MM, Harlaar J. Effects of adding a virtual reality environment to different modes of treadmill walking. Gait & posture. 2014;39(3):939–45. doi: 10.1016/j.gaitpost.2013.12.005 . 24412269 10.1016/j.gaitpost.2013.12.005

[16] Liu WY, Meijer K, Delbressine JM, Willems PJ, Franssen FM, Wouters EF, et al Reproducibility and Validity of the 6-Minute Walk Test Using the Gait Real-Time Analysis Interactive Lab in Patients with COPD and Healthy Elderly. PloS one. 2016;11(9):e0162444 doi: 10.1371/journal.pone.0162444 ; PubMed Central PMCID: PMC5015964. 27607426 10.1371/journal.pone.0162444 PMC5015964

[17] Helbostad JL, Moe-Nilssen R. The effect of gait speed on lateral balance control during walking in healthy elderly. Gait & posture. 2003;18(2):27–36. . 14654205 10.1016/s0966-6362(02)00197-2

[18] Rutten EP, Gopal P, Wouters EF, Franssen FM, Hageman GJ, Vanfleteren LE, et al Various Mechanistic Pathways Representing the Aging Process Are Altered in COPD. Chest. 2016;149(1):53–61. doi: 10.1378/chest.15-0645 . 26066545 10.1378/chest.15-0645

[19] Borges O. Isometric and isokinetic knee extension and flexion torque in men and women aged 20–70. Scandinavian journal of rehabilitation medicine. 1989;21(1):45–53. . 2711137

[20] van den Bogert AJ, Geijtenbeek T, Even-Zohar O, Steenbrink F, Hardin EC. A real-time system for biomechanical analysis of human movement and muscle function. Medical & biological engineering & computing. 2013;51(10):1069–77. doi: 10.1007/s11517-013-1076-z ; PubMed Central PMCID: PMC3751375. 23884905 10.1007/s11517-013-1076-z PMC3751375

[21] Holland AE, Spruit MA, Troosters T, Puhan MA, Pepin V, Saey D, et al An official European Respiratory Society/American Thoracic Society technical standard: field walking tests in chronic respiratory disease. The European respiratory journal: official journal of the European Society for Clinical Respiratory Physiology. 2014;44(6):1428–46. doi: 10.1183/09031936.00150314 . 25359355 10.1183/09031936.00150314

[22] Butcher SJ, Meshke JM, Sheppard MS. Reductions in functional balance, coordination, and mobility measures among patients with stable chronic obstructive pulmonary disease. Journal of cardiopulmonary rehabilitation. 2004;24(4):274–80. Epub 2004/08/03. . 15286536 10.1097/00008483-200407000-00013

[23] Menard-Rothe K, Sobush DC, Bousamra M 2nd, Haasler, Lipchik RJ. Self-selected walking velocity for functional ambulation in patients with end-stage emphysema. Journal of cardiopulmonary rehabilitation. 1997;17(2):85–91. Epub 1997/03/01. . 9101385 10.1097/00008483-199703000-00003

[24] Roig M, Eng JJ, MacIntyre DL, Road JD, FitzGerald JM, Burns J, et al Falls in people with chronic obstructive pulmonary disease: an observational cohort study. Respiratory medicine. 2011;105(3):461–9. doi: 10.1016/j.rmed.2010.08.015 ; PubMed Central PMCID: PMC3350813. 20869227 10.1016/j.rmed.2010.08.015 PMC3350813

[25] Mador MJ, Krauza M, Shaffer M. Effect of exercise training in patients with chronic obstructive pulmonary disease compared with healthy elderly subjects. Journal of cardiopulmonary rehabilitation and prevention. 2012;32(3):155–62. doi: 10.1097/HCR.0b013e31824e16e4 . 22487615 10.1097/HCR.0b013e31824e16e4

[26] Roig M, Eng JJ, Road JD, Reid WD. Falls in patients with chronic obstructive pulmonary disease: a call for further research. Respiratory medicine. 2009;103(9):1257–69. Epub 2009/05/08. doi: 10.1016/j.rmed.2009.03.022 ; PubMed Central PMCID: PMC3326069. 19419852 10.1016/j.rmed.2009.03.022 PMC3326069

[27] Mbourou GA, Lajoie Y, Teasdale N. Step length variability at gait initiation in elderly fallers and non-fallers, and young adults. Gerontology. 2003;49(1):21–6. doi: 66506. doi: 10.1159/000066506 . 12457046 10.1159/000066506

[28] Hausdorff JM, Edelberg HK, Mitchell SL, Goldberger AL, Wei JY. Increased gait unsteadiness in community-dwelling elderly fallers. Archives of physical medicine and rehabilitation. 1997;78(3):278–83. . 9084350 10.1016/s0003-9993(97)90034-4

[29] Gabell A, Nayak US. The effect of age on variability in gait. Journal of gerontology. 1984;39(6):662–6. . 6491179 10.1093/geronj/39.6.662

[30] Maki BE. Gait changes in older adults: predictors of falls or indicators of fear. Journal of the American Geriatrics Society. 1997;45(3):313–20. . 9063277 10.1111/j.1532-5415.1997.tb00946.x

[31] Hausdorff JM. Gait dynamics, fractals and falls: finding meaning in the stride-to-stride fluctuations of human walking. Human movement science. 2007;26(4):555–89. Epub 2007/07/10. doi: 10.1016/j.humov.2007.05.003 ; PubMed Central PMCID: PMC2267927. 17618701 10.1016/j.humov.2007.05.003 PMC2267927

[32] Hausdorff JM, Mitchell SL, Firtion R, Peng CK, Cudkowicz ME, Wei JY, et al Altered fractal dynamics of gait: reduced stride-interval correlations with aging and Huntington's disease. J Appl Physiol (1985). 1997;82(1):262–9. doi: 10.1152/jappl.1997.82.1.262 . 9029225 10.1152/jappl.1997.82.1.262

[33] Toebes MJ, Hoozemans MJ, Furrer R, Dekker J, van Dieen JH. Local dynamic stability and variability of gait are associated with fall history in elderly subjects. Gait & posture. 2012;36(3):527–31. doi: 10.1016/j.gaitpost.2012.05.016 . 22748312 10.1016/j.gaitpost.2012.05.016

[34] Stergiou N, Harbourne R, Cavanaugh J. Optimal movement variability: a new theoretical perspective for neurologic physical therapy. Journal of neurologic physical therapy: JNPT. 2006;30(3):120–9. . 17029655 10.1097/01.npt.0000281949.48193.d9

[35] Gea J, Pascual S, Casadevall C, Orozco-Levi M, Barreiro E. Muscle dysfunction in chronic obstructive pulmonary disease: update on causes and biological findings. Journal of thoracic disease. 2015;7(10):E418–38. doi: 10.3978/j.issn.2072-1439.2015.08.04 ; PubMed Central PMCID: PMC4635259. 26623119 10.3978/j.issn.2072-1439.2015.08.04 PMC4635259

[36] Barreiro E, Bustamante V, Cejudo P, Galdiz JB, Gea J, de Lucas P, et al Guidelines for the evaluation and treatment of muscle dysfunction in patients with chronic obstructive pulmonary disease. Archivos de bronconeumologia. 2015;51(8):384–95. doi: 10.1016/j.arbres.2015.04.011 . 26072153 10.1016/j.arbres.2015.04.011

[37] Donaldson AV, Maddocks M, Martolini D, Polkey MI, Man WD. Muscle function in COPD: a complex interplay. International journal of chronic obstructive pulmonary disease. 2012;7:523–35. doi: 10.2147/COPD.S28247 ; PubMed Central PMCID: PMC3430120. 22973093 10.2147/COPD.S28247 PMC3430120

[38] Agrawal MB, Awad NT. Correlation between Six Minute Walk Test and Spirometry in Chronic Pulmonary Disease. Journal of clinical and diagnostic research: JCDR. 2015;9(8):OC01–4. doi: 10.7860/JCDR/2015/13181.6311 ; PubMed Central PMCID: PMC4576573. 26435980 10.7860/JCDR/2015/13181.6311 PMC4576573

[39] Wijkstra PJ, TenVergert EM, van der Mark TW, Postma DS, Van Altena R, Kraan J, et al Relation of lung function, maximal inspiratory pressure, dyspnoea, and quality of life with exercise capacity in patients with chronic obstructive pulmonary disease. Thorax. 1994;49(5):468–72. ; PubMed Central PMCID: PMC474868. 8016768 10.1136/thx.49.5.468 PMC474868

[40] Gosker HR, Wouters EF, van der Vusse GJ, Schols AM. Skeletal muscle dysfunction in chronic obstructive pulmonary disease and chronic heart failure: underlying mechanisms and therapy perspectives. The American journal of clinical nutrition. 2000;71(5):1033–47. . 10799364 10.1093/ajcn/71.5.1033

[41] Gosselink R, Troosters T, Decramer M. Peripheral muscle weakness contributes to exercise limitation in COPD. American journal of respiratory and critical care medicine. 1996;153(3):976–80. doi: 10.1164/ajrccm.153.3.8630582 . 8630582 10.1164/ajrccm.153.3.8630582

[42] Liu MQ, Anderson FC, Schwartz MH, Delp SL. Muscle contributions to support and progression over a range of walking speeds. Journal of biomechanics. 2008;41(15):3243–52. doi: 10.1016/j.jbiomech.2008.07.031 ; PubMed Central PMCID: PMC4423744. 18822415 10.1016/j.jbiomech.2008.07.031 PMC4423744

[43] Murray MP, Guten GN, Sepic SB, Gardner GM, Baldwin JM. Function of the triceps surae during gait. Compensatory mechanisms for unilateral loss. The Journal of bone and joint surgery American volume. 1978;60(4):473–6. . 670268

[44] Requiao LF, Nadeau S, Milot MH, Gravel D, Bourbonnais D, Gagnon D. Quantification of level of effort at the plantarflexors and hip extensors and flexor muscles in healthy subjects walking at different cadences. Journal of electromyography and kinesiology: official journal of the International Society of Electrophysiological Kinesiology. 2005;15(4):393–405. doi: 10.1016/j.jelekin.2004.12.004 . 15811610 10.1016/j.jelekin.2004.12.004

[45] Sutherland DH, Cooper L, Daniel D. The role of the ankle plantar flexors in normal walking. The Journal of bone and joint surgery American volume. 1980;62(3):354–63. . 7364808

[46] Gagnon P, Maltais F, Bouyer L, Ribeiro F, Coats V, Brouillard C, et al Distal leg muscle function in patients with COPD. Copd. 2013;10(2):235–42. doi: 10.3109/15412555.2012.719047 . 23547635 10.3109/15412555.2012.719047

[47] Maddocks M, Jones M, Snell T, Connolly B, de Wolf-Linder S, Moxham J, et al Ankle dorsiflexor muscle size, composition and force with ageing and chronic obstructive pulmonary disease. Experimental physiology. 2014;99(8):1078–88. doi: 10.1113/expphysiol.2014.080093 . 24928952 10.1113/expphysiol.2014.080093

[48] Lee SJ, Hidler J. Biomechanics of overground vs. treadmill walking in healthy individuals. J Appl Physiol (1985). 2008;104(3):747–55. Epub 2007/12/01. doi: 10.1152/japplphysiol.01380.2006 . 18048582 10.1152/japplphysiol.01380.2006

[49] Riley PO, Paolini G, Della Croce U, Paylo KW, Kerrigan DC. A kinematic and kinetic comparison of overground and treadmill walking in healthy subjects. Gait & posture. 2007;26(1):17–24. doi: 10.1016/j.gaitpost.2006.07.003 . 16905322 10.1016/j.gaitpost.2006.07.003

[50] Alton F, Baldey L, Caplan S, Morrissey MC. A kinematic comparison of overground and treadmill walking. Clinical biomechanics. 1998;13(6):434–40. . 11415818 10.1016/s0268-0033(98)00012-6

[51] Sloot LH, van der Krogt MM, Harlaar J. Self-paced versus fixed speed treadmill walking. Gait & posture. 2013 Epub 2013/09/24. doi: 10.1016/j.gaitpost.2013.08.022 . 24055003 10.1016/j.gaitpost.2013.08.022

