# Supplementary material for: Spatiotemporal gait characteristics in patients with COPD during the Gait Real-time Analysis Interactive Lab-based 6-minute walk test
Source: PLoS One. 2017 Dec 28;12(12):e0190099. doi: 10.1371/journal.pone.0190099 (PMC5746246; doi:10.1371/journal.pone.0190099)
Supplement: S1 Appendix — (PDF) [file pone.0190099.s001.pdf]

S1 Appendix. Data processing

Walking speed of fifteen patients with COPD and fifteen healthy elderly were analysed to assess the duration of acceleration and deceleration of walking speed of subjects during the GRAIL-based 6MWT. Subjects needed time to get accustomed to split-belt, self-paced treadmill walking. Patients with COPD seemed to need a longer period to achieve their preferred walking speed to perform the 6MWT compared to healthy elderly (Figure A and B in S1 Appendix). Therefore, the first minute was removed for analysis. According to the ERS/ATS statement, subjects will be informed that the 6MWT is about to end [1]. To avoid deceleration influencing gait characteristics 15 seconds prior to the end of the 6MWT will be removed for analysis. Therefore, data were cropped from 60 seconds to 345 seconds, to minimize start-up and deceleration effects.

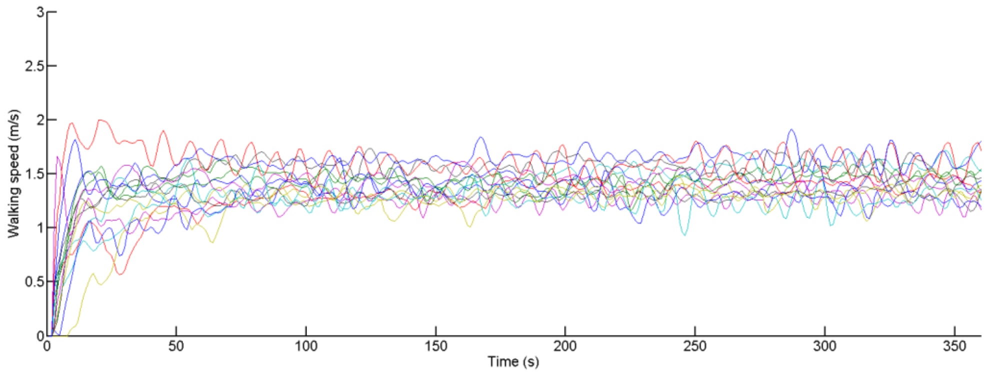

Figure A. Walking speed of patients with COPD during the GRAIL-based 6MWT

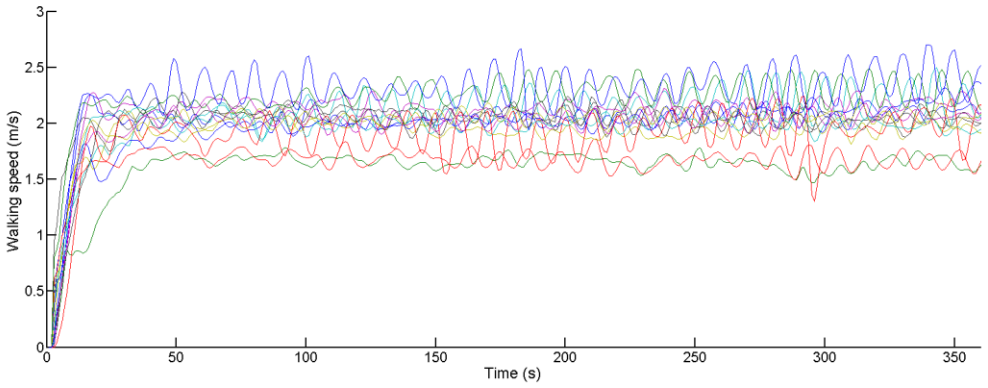

Figure B. Walking speed of healthy elderly subjects during the GRAIL-based 6MWT

1. Holland AE, Spruit MA, Troosters T, Puhan MA, Pepin V, et al. (2014) An official European Respiratory Society/American Thoracic Society technical standard: field walking tests in chronic respiratory disease. Eur Respir J 44: 1428-1446.
