# Supplementary material for: Spatiotemporal gait characteristics in patients with COPD during the Gait Real-time Analysis Interactive Lab-based 6-minute walk test
Source: PLoS One. 2017 Dec 28;12(12):e0190099. doi: 10.1371/journal.pone.0190099 (PMC5746246; doi:10.1371/journal.pone.0190099)
Supplement: S2 Appendix — (PDF) [file pone.0190099.s002.pdf]

S2 Appendix. Data correction and event detection

All gaps were reconstructed using a spline interpolation in MATLAB (MathWorks Inc., Natick, USA). The tracks were then filtered using a low pass second order Butterworth filter, with a cut-off frequency at 12 Hz. All gaps, exceeding 3 samples at the start and end, were reconstructed using the filtered tracks.

Gait events were calculated using the treadmill velocity based method of Zeni et al. [1]. Foot marker position data were filtered using a second order Butterworth filter, with a cut-off frequency of 10 Hz, before velocity was calculated. Gait events were determined based on force plate data [2-4], using a 50 Newton threshold. The phase shift between the results of both methods was determined for steps in which the force exceeded the threshold. Outliers were detected and removed using a number of samples threshold and when the phase shift exceeded a value of more than 2 times the standard deviation. All events, calculated with the velocity based method, were then corrected with the calculated phase shift. Then spatiotemporal gait characteristics were determined using a custom Matlab program.

1. Zeni JA, Jr., Richards JG, Higginson JS (2008) Two simple methods for determining gait events during treadmill and overground walking using kinematic data. Gait Posture 27: 710-714.
2. Ghoussayni S, Stevens C, Durham S, Ewins D (2004) Assessment and validation of a simple automated method for the detection of gait events and intervals. Gait Posture 20: 266-272.
3. Hansen AH, Childress DS, Meier MR (2002) A simple method for determination of gait events. J Biomech 35: 135-138.
4. Hreljac A, Marshall RN (2000) Algorithms to determine event timing during normal walking using kinematic data. J Biomech 33: 783-786.
