# Supplementary material for: Spatiotemporal gait characteristics in patients with COPD during the Gait Real-time Analysis Interactive Lab-based 6-minute walk test
Source: PLoS One. 2017 Dec 28;12(12):e0190099. doi: 10.1371/journal.pone.0190099 (PMC5746246; doi:10.1371/journal.pone.0190099)
Supplement: S3 Appendix — (PDF) [file pone.0190099.s003.pdf]

S3 Appendix. Heterogeneity in COPD and healthy elderly group

The patient group seems to be more heterogeneous in the variability of temporal gait characteristics, stride length and step length compared to healthy elderly (table 3), due to the increased standard deviation of these gait characteristics in the patient group for both analyses. Extreme values in mean gait characteristics were more prone in the patient group (>1.5 interquartile range: 13 patients and 3 healthy elderly; >3.0 interquartile range: 1 patient). Seventeen patients with COPD could be regarded as extreme values (>1.5 interquartile range) in contrast to 8 healthy elderly subjects. Four patients had interquartile ranges above 3.0 compared to 2 healthy elderly. All subjects were included for analysis, as these values represent the heterogeneity of the subjects in each group.
