# Supplementary material for: Spatiotemporal gait characteristics in patients with COPD during the Gait Real-time Analysis Interactive Lab-based 6-minute walk test
Source: PLoS One. 2017 Dec 28;12(12):e0190099. doi: 10.1371/journal.pone.0190099 (PMC5746246; doi:10.1371/journal.pone.0190099)
Supplement: S5 Appendix — (PDF) [file pone.0190099.s005.pdf]

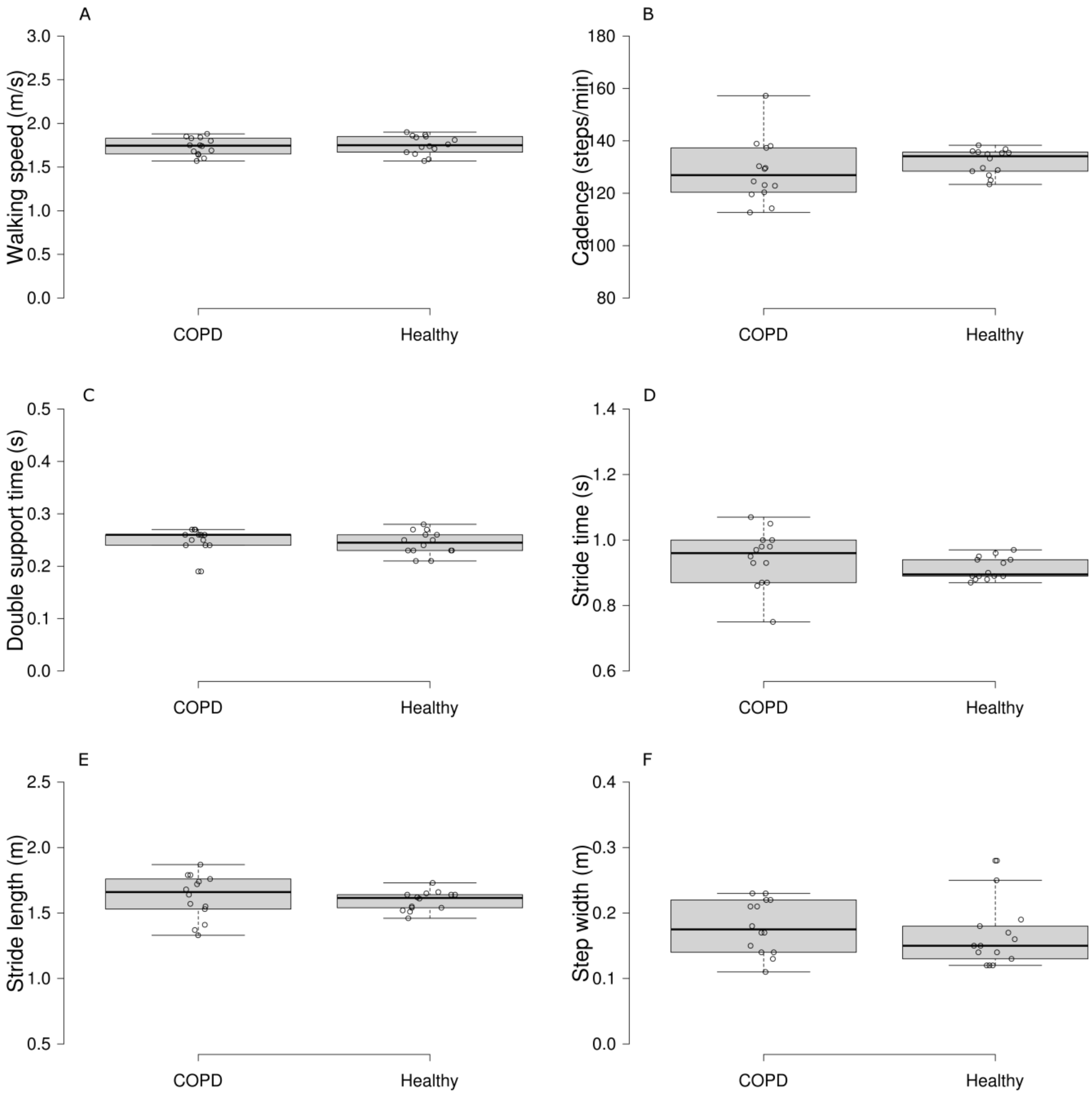

Figure A. Boxplots of spatiotemporal gait characteristics in the sub-analysis. Patients with COPD (n=14) and healthy elderly (n=14) are depicted. Centre lines show the medians; box limits indicate the 25th and 75th percentiles; whiskers extend 1.5 times the interquartile range from the 25th and 75th percentiles, outliers are represented by dots.

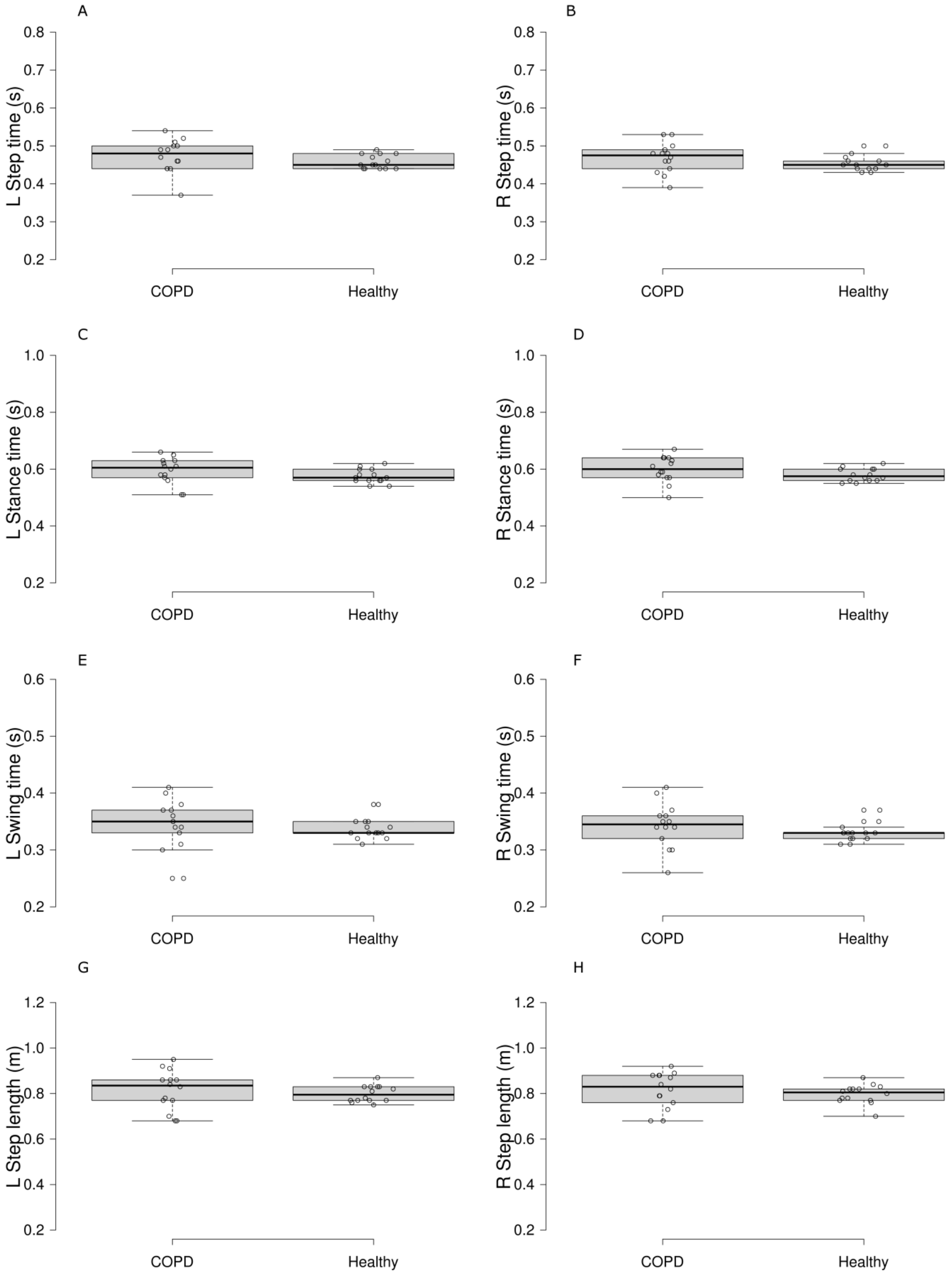

Figure B. Boxplots of spatiotemporal gait characteristics separated for left and right side in the sub-analysis. Patients with COPD (n=14) and healthy elderly (n=14) are depicted. Centre lines show the medians; box limits indicate the 25th and 75th percentiles; whiskers extend 1.5 times the interquartile range from the 25th and 75th percentiles, outliers are represented by dots.
